# Training novice practitioners to reliably report their meditation experience using shared phenomenological dimensions

**DOI:** 10.1016/j.concog.2019.01.004

**Published:** 2019-02

**Authors:** Oussama Abdoun, Jelle Zorn, Stefano Poletti, Enrico Fucci, Antoine Lutz

**Affiliations:** Lyon Neuroscience Research Centre, INSERM U1028, CNRS UMR5292, Lyon 1 University, Lyon, France

**Keywords:** Meditation, Mindfulness, Phenomenology, Neurophenomenology, Self-reports, First-person, Training, Demand characteristics, Desirable responding

## Abstract

Empirical descriptions of the phenomenology of meditation states rely on practitioners’ ability to provide accurate information on their experience. We present a meditation training protocol that was designed to equip naive participants with a theoretical background and experiential knowledge that would enable them to share their experience. Subsequently, novices carried on with daily practice during several weeks before participating in experiments. Using a neurophenomenological experiment designed to explore two different meditation states (focused attention and open monitoring), we found that self-reported phenomenological ratings (i) were sensitive to meditation states, (ii) reflected meditation dose and fatigue effects, and (iii) correlated with behavioral measures (variability of response time). Each of these effects was better predicted by features of participants’ daily practice than by desirable responding. Our results provide evidence that novice practitioners can reliably report their experience along phenomenological dimensions and warrant the future investigation of this training protocol with a longitudinal design.

## Highlights

• Novice meditators can be trained to report their phenomenological experience.
• Self-reports are associated with history of practice, not with desirable responding.
• Balance between focused attention (FA) and open monitoring (OM) practices predicts the ability to dissociate them.
• Daily practice of FA predicts the felt stability of long sessions.
• Total practice of OM predicts phenomenological proficiency.

## 1 Introduction

This article aims to describe a meditation training protocol developed in the context of an empirical brain imaging, cross-sectional study that investigates the mechanisms of mindfulness and compassion meditations. The novelty of this protocol is to obtain a meditation active control group by training healthy, naive participants to verbally express their subjective experience of meditation practice using a multidimensional phenomenological space (Lutz, Jha, Dunne, & Saron, 2015). Phenomenological space refers here to the description of features of the field of experience, as it is lived and verbally expressed in the first person (e.g., Husserl, 1991). This phenomenological matrix has been recently proposed as a framework to map different styles and levels of training in mindfulness, as well as heuristic tool to generate hypotheses for empirical research.

The *Brain & Mindfulness* project attempts to practically apply this theoretical framework (for the study manual, see Abdoun, Zorn, Fucci, Perraud, Aarts, & Lutz, 2018). During the training participants were introduced to various styles of meditation practices and acquainted with phenomenological categories through various experiential exercises. These phenomenological dimensions were then investigated at neural, behavioral and physiological levels during the various cognitive and affective experimental paradigms. Such explicit use of first-person data to guide the analysis of third-person data is inspired by Francisco Varela’s research program of neurophenomenology (Lutz and Thompson, 2003, Varela, 1996).

The current training protocol attempts to pragmatically tackle three methodological and conceptual challenges. The first one is concerned with issues regarding the definition of mindfulness meditation in psychology and cognitive neuroscience. The second one pertains to epistemological and methodological issues related to the integration of first-person reports in an experimental protocol. The third one is related to the quality of control groups for cross-sectional studies of meditation expertise.

### 1.1 Theoretical context: mindfulness as a dimensional, phenomenological state

In experimental and clinical psychology, the construct of mindfulness is generally used with three different meanings that refer either to: (a) a mental trait or a dispositional inclination (e.g. the Five Facet of Mindfulness proposed by Baer, Smith, Hopkins, Krietemeyer, & Toney, 2006), (b) a soteriological or spiritual path conceived in therapeutic and health-promotion terms (e.g. in the Mindfulness-Based-Stress-Reduction program; Kabat-Zinn, 1982), and (c) a single cognitive process trained and potentially brought to various human activities (e.g. like in “paying attention in a particular way: on purpose, in the present moment, and non-judgmentally”, Kabat-Zinn, 1994, p.4; or “the optimal interaction between attention and peripheral awareness”, Culadasa et al., 2015, p.30). While these meanings remain useful for many contexts, they are also problematic. Self-report questionnaires to study mindfulness as a trait lack specificity (Goldberg et al., 2016) and may even yield contradictory findings. For instance, Leigh, Bowen, and Marlatt (2005) found that binge drinkers’ mindfulness scores were higher than those of participants in a mindfulness retreat. In addition, findings may be biased by social desirability, consistency effects, or shared language between intervention instructions and scales (see Sauer et al., 2013, Van Dam et al., 2012). Interpreting mindfulness as a soteriological process (meaning [b]) is often too broad to guide empirical research. Up to this point, discussions of mindfulness as a cognitive process (meaning [c]) make it difficult to account for differences in practice styles and levels of expertise, while also lacking the specificity required to formulate mechanistic hypotheses. Because these meanings are too restrictive, with C. Saron, A. Jha and J Dunne, we have argued against formulating a single, universally applicable consensus definition of mindfulness (Lutz et al., 2015).

Instead we favor reconceiving mindfulness through a family resemblance approach whereby it can be conceptualized as “a variety of cognitive processes embedded in a complex postural, aspirational, and motivational context that contribute to states that resemble one another along well-defined phenomenological dimensions” (Lutz et al., 2015, p.633). This approach draws on previous efforts to conceptualize mindfulness (Chambers et al., 2009, Hölzel et al., 2011, Lutz et al., 2008) and the phenomenology of mindfulness practice. It is compatible with multiple explanatory and analytical frameworks from different subdisciplines, including contemplative theories, clinical frameworks and psychological and neuroscientific models. This approach is guided by a pragmatic inquiry: when one is formally practicing mindfulness, what observable and manipulable features of consciousness are most relevant to report in an experimental setting? We identified seven features proposed in a bipartite phenomenological model (detailed in Lutz et al., 2015 and resumed here in Table 1).

**Table 1:** The seven phenomenological dimensions proposed in Lutz et al. (2015). Although primary dimensions are presented in an orthogonal Euclidean space, they can vary independently from one another. Within this multidimensional space, secondary dimensions represent features dependent on specific mental states and level of expertise. In addition, the model assumes four general features that are common across the family of practices associated to mindfulness, that are physical posture, non-aversive affect, axiological framework, and task-set maintenance. These common general features are necessary elements of mindfulness practice, but they are not explicitly depicted in the model because they are less significant in distinguishing styles of practice. In the present manuscript, these general features will not be explicitly discussed, even if they were measured during the experimental settings. For instance, we measure non-aversive affect dimension during a nociceptive paradigm, and we interviewed after this paradigm the participants about the relationship between pain and their worldview.

Primary dimensions	Secondary dimensions
**Object orientation** Sense that a mental state is oriented towards some object or class of objects (e.g. perceptions, emotions)	**Aperture** Broadness of the scope of attention **Clarity** Degree of vividness of the experience

**Dereification** Degree to which mental phenomena are experienced as mental processes rather than accurate depictions of reality	**Stability** Degree to which experience presents itself as enduring over time

**Meta-Awareness** Extent to which the experience is under monitoring	**Effort** Impression that one’s present mental state is easy or difficult to maintain

The model assumes that these dimensions of experience are dynamic and manipulable in that they are affected—directly or indirectly—by different instructions of practice and/or by the level of expertise. This model was used to plot the hypothetical phenomenological characteristics of two styles of mindfulness, Focused attention (FA) and Open monitoring (OM) meditations, for both novice and expert meditators (Lutz et al., 2015). These plots have been created based on various instruction sets and descriptions. They should not be taken as actual plots of any individual’s phenomenology. The same set of mindfulness instructions could be mapped to different points in the phenomenological space. This is due to individual differences between practitioners in the manner in which they interpret and instantiate instructions.

One aim of the Brain & Mindfulness project is to implement this heuristic model and to empirically test some of its assumptions. For instance, can we use self-report scales to reliably measure and monitor consistent changes in these features in response to different meditation instructions and training, congruent with the hypothetical plots previously published?

### 1.2 Epistemological limitations: Reliability of self-report data

A second methodological aim arises from the first one: are the empirically-obtained plots of the different styles and levels of expertise reliable in this phenomenological space?

#### 1.2.1 The reliability of self-reports

The perceived demise of early twentieth century introspectionism (Costall, 2006) and the seminal review by Nisbett and Wilson (1977) questioned the ability of the participants to report the real causes of their behavior. Since then, introspective-like methods have been looked upon with distrust by many in the fields of psychology and cognitive science. Contrastingly, others have warned against drawing general conclusions from these failures. For example, Hurlburt and Heavey (2001) have criticized how Nisbett and Wilson’s work has been carelessly taken as “an unconditional refutation of introspection in general, not merely of the attribution of causation”, thus ignoring that “even Nisbett and Wilson recognized the possibility of accurate reports about inner experience” (Hurlburt & Heavey, 2001, p.401).

Devising ways to detect and/or limit the diverse types of self-reports distortions is an active field of methodological research. For example, self-administered questionnaires have long included validity scales designed to this effect (Baer, Rinaldo, & Berry, 2003). More recently, there has been a renewed interest for ‘first-person methods’ to study consciousness (see the three special issues of the *Journal of Consciousness Studies* on this question: Jack and Roepstorff, 2003, Jack and Roepstorff, 2004, Hasenkamp and Thompson, 2013). First-person methods refer to methods that allow an investigator to bring a participant close to their subjective experience^1^ (Petitmengin, 2006), as well as to practices that subjects themselves can use to increase their sensitivity to their own experiences (Bitbol and Petitmengin, 2013, Depraz et al., 2003, Petitmengin et al., 2013, Varela and Shear, 1999).

Meditation training has been proposed as a pragmatic response to this challenge due to it's disciplined approach to examining experience. Approaching experience from this perspective allows for the refinement of first person categories' repertoire and strengthen the robustness of the relationship between first and third-person data (Varela, 1996). However, this hypothesis remains to be thoroughly tested. Current available evidence includes the improvement of the congruence between implicit and explicit measures of self-views after brief mindfulness exercises (see Strick & Papies, 2017 for a study on affiliation motives and goals, and Koole, Govorun, Cheng, & Gallucci, 2009 for a study on self-esteem). In contrast, measures of interoceptive awareness based on heartbeat perception in experienced meditators have yielded mixed and contradictory results (Bornemann and Singer, 2017, Khalsa et al., 2008, Melloni et al., 2013). The inconclusiveness of these studies may be due to a lack of methodological validity (Zamariola, Maurage, Luminet, & Corneille, 2018), discrepancies in the experimental designs and/or in the extent of bodily focus in participants’ meditation practice.

#### 1.2.2 Demand characteristics and desirable responding

In the context of phenomenological research on self-induced mental states (such as in meditation research), demand characteristics is a major source of confound that undermines the credibility of self-reports. *Demand characteristics* refer to “the totality of cues which convey an experimental hypothesis to the subject[s]” and which consequently “become significant determinants of subjects' behavior” (Orne, 1962, p.779). Participants volunteering for scientific experiments have various motivations that may, consciously or unconsciously, incite them to play the role of the good participant and try to serve the experiment by producing the data that they think will confirm the (presumed) research hypothesis. To attenuate the confounding effects of demand characteristics, researchers commonly resort to the concealment of – if not the deception about – hypotheses, manipulations, dependent measures and independent variables. Another source of distortion of a participant’s behavior is his/her wish to present herself favorably to the experimenter, who may be perceived as an evaluator. This so-called *social desirability* bias is related to the effect of demand characteristics, but not identical to it (Weber & Cook, 1972). To eliminate this confound, some researchers advocate the use of scales developed to capture individuals’ inclination to self-enhancement (Crowne and Marlowe, 1960, Paulhus, 1984), as covariates in the models assessing the effects of interest.

In the phenomenological study of meditation, demand characteristics lurk in the large overlap between the semantics of meditation instructions taught or familiar to the participants, and the phrasing of self-report scales aimed at measuring the phenomenological dimensions of interest (e.g. terms such as present-centered and nonjudgmental, see Van Dam et al., 2012). Consequently, the magnitude of self-reported phenomenological features of meditation remain overshadowed by doubt, even when shown to be highly specific (see for example Kok & Singer, 2017). Unfortunately, the usual concealment strategies can hardly be applied in this context, considering that participants are necessarily aware of the manipulation in so far as they are asked to implement it through the practice of meditation.

This is not to say that all self-report results from meditation studies are inexorably confounded by the effect of demand characteristics. Even when demand characteristics are difficult to attenuate, one can look for evidence that supports an interpretation of the effect beyond their impact. In this study, we adopt a strategy of comparing certain factors (which are unaffected by demand characteristics or desirable responding) with self-reporting effects. We will illustrate this general approach in two ways: (i) within-subject, by testing whether fluctuations of phenomenological self-reports correlate with relevant behavioral measures, and (ii) across subjects, by testing whether participants’ amount and structure of daily practice predict the self-reported effects on phenomenological dimensions.

### 1.3 Methodological issue: quality active control group

A critical effort of the *Brain & Mindfulness* project was to refine the matching between the control group and expert meditators. This was done primarily by training novices in different styles of meditation practice and by familiarizing them to different phenomenological categories of interest. A prominent issue in the field of neuroscientific studies on mindfulness meditation is the relative paucity of high quality active control (Goldberg et al., 2017). This issue has been repeatedly raised and suggestions of improvement have been discussed in the context of longitudinal studies (Davidson and Kaszniak, 2015, Kuyken et al., 2016). However, cross-sectional studies with long term practitioners have received less methodological attention. In such studies, an active control group is often lacking or too basic when present. Of the nineteen independent cross-sectional studies on the neurofunctional effects of long term meditation in a recent meta-analysis (Fox et al., 2016), only seven included an active control.^2^ In six of these studies, meditation-naive participants received written and/or oral instructions and were encouraged to sustain a daily practice for 7 to 10 days until the moment of the experiment. In the remaining study, participants received a brief training session by an experienced teacher on the day of the experiment (Kalyani et al., 2011). These approaches, while clearly better than not including an active control group, have several limitations. First, limited possibility for feedback or the lack of guidance by an experienced and qualified instructor induces a high risk of misinterpretations and inadequate implementations of the practices. Second, the short duration of the training limits the opportunities to engage with the practice. Here we addresses some of these issues by: (1) formally training meditation-naive participants in practices from the same meditation background as the long-term practitioners, (2) letting this training be provided by a qualified instructor in a context with attention to sufficient opportunity for guided practice and feedback, and (3) encouraging participants to maintain a daily practice at home for a minimum of 20 min a day for an extended duration (6–22 weeks depending on the availability of participants and experimental resources). We contend that these adaptations make it more likely for participants to reach a refined understanding of the various practices and experiential dimensions at hand. This training has the potential of reducing the risk that any group differences are merely driven by confounding factors (e.g. misunderstanding practices and/or unfamiliarity with meditation terminology for novices but not experts), instead of reflecting the true effect of interest, i.e. meditation expertise.

We will first report the specifics of our meditation training protocol. Then we will provide empirical evidence for its effectiveness in teaching meditation-naive participants to use first-person categories to describe their conscious experience and discriminate between phenomenological dimensions.

## 2 Methods

### 2.1 Participants

The first stage of the research included a meditation training weekend comprising of 42 healthy participants naive to meditation. These individuals were recruited for their interest to learn meditation and their willingness to sustain a regular practice for several months. After a preliminary inclusion procedure (see study manual for details, Abdoun et al., 2018), participants were invited to attend a weekend-long training program in the Lyon Neuroscience Research Center. The program looked to support participants in developing a refined understanding of the states of consciousness involved in the following experimental study.

The expert group was comprised of 30 healthy long-term practitioners with more than 10,000 h of formal meditation in their life and trained in the Kagyü and Nyingma schools of Tibetan Buddhism.

Both novices and experts participated in up to 8 experimental sessions (see study manual for details, Abdoun et al., 2018). For expert participants, these experimental sessions were gathered in 2 visits of 3 days, or a single visit of 6 days. For novice participants, each visit comprised 1 or 2 experimental sessions, and the visits were spread over a period spanning from 2 to 23 weeks after the training weekend. Visits were scheduled according to participants’ and equipment (MRI, MEG, EEG) availabilities, leading to a large but quasi-random variability across the novice group in time elapsed between the training and the experiment.

Among all participants, 25 trained novice practitioners and 25 expert practitioners participated in the MEG experiment described below. The remaining participants included in the larger study were excluded for the MEG experiment because of excessive signal artifacts caused by dental prostheses. The novice and expert groups for the MEG experiment did not differ in gender (16 and 15 males, respectively; χ^2^(1) = 0.08), age (53.9 ± 7.1 and 51.6 ± 8.0 years, respectively; independent *t*-tests t = 0.84) and education (3.88 ± 2.15 and 3.20 ± 2.16 years of higher education, respectively; independent *t*-tests t = 0.46).

### 2.2 Meditation training protocol

#### 2.2.1 General outline

The training protocol was based on *Joy of Living* (Rinpoche and Swanson, 2007, Tergar, n.d.), a secular meditation program aimed at Western audiences authored by Yongey Mingyur Rinpoche, a renowned master of Karma Kagyü and Nyingma schools of Tibetan Buddhism. This program was selected for its shared background with experts’ training. In its original format, the program is divided into three stages, each lasting two days; in addition, there are minimal practice requirements to attend stages 2 and 3. For our training protocol, we drew from the material of stages 1 and 2, condensing them in a two-day format, and included adaptations to emphasize the specific dimensions of experience of interest to the research program.

The training was provided by a qualified instructor with thirteen years of practice under the guidance of Mingyur Rinpoche, and eight years of teaching experience with the *Joy of Living* program. The training included teachings with the support of instruction videos, guided meditations and experiential exercises, question and answer sessions, as well as sufficient time to reflect and share within the group.

The training allowed a basic understanding of a few selected phenomenological dimensions eligible for an active comparison with expert practitioners. In particular, the program introduced participants to the following dimensions: effort, aperture, absorption vs. meditative awareness, foreground vs. background awareness, equanimity, clarity (see Table 1 and supplementary materials). The discernment of these dimensions was implemented by introducing the lived phenomenology of these states and creating occasions for a direct exploration of them. To access both the experience and the meaning of meditation the teacher devised specific exercises with connected theoretical principles. As a sommelier apprentice does in tasting, savoring, comparing and sampling different wines under the guidance of a sommelier, participants were invited to learn, practice and distinguish few states of consciousness under the guidance of a meditation teacher in order to become progressively familiar with some meditative phenomenological dimensions commonly described in contemplative traditions.

The training followed a specific day program (Table 2) which will be briefly described here.

**Table 2:** Program of the training weekend.

DAY 1	DAY 2
**11:00 – 12:00** Effort in perception and attention (*on a sound*) Absorption vs. Meditative awareness (*introduction*)	**9:30 – 10:30** Meditative awareness
	**BREAK**

**12:00 – 13:00** Absorption vs. Meditative awareness (*on the breath*)	**10:45 – 12:30** Group discussion Empathy vs. Compassion (*introduction*)

**BREAK**	**BREAK**

**14:30 – 15:30** Absorption vs. Meditative awareness (*on the breath*)	**14:00 – 15:45** Focused Attention & Open Monitoring (*on pain*) Empathy vs. Compassion (*images*)

**BREAK**	**BREAK**

**16:30 – 17:30** Focused Attention & Open Monitoring Object orientation & Aperture Background & Foreground	**16:00 – 17:00** Closing meditation (intention to practice) Presentation of the research project Instructions for home practice

In **day 1**, participants were first introduced to the notion of mental *‘effort’* in meditation through an experiential exercise that involved listening to sounds. The rest of the day was spent exploring the concepts of *‘absorption and meditative awareness’*. This exercise was done first by using the breath as an anchor for meditative awareness. Participants were asked to restrict their attention to the breath, to notice when their mind had wandered, and to return their attention to the breath when this happened. Later during the day, they were also asked to gradually explore more open forms of awareness, by opening up to sense experiences from the environment (e.g. sounds and vision). While doing so, participants also engaged in two other experiential exercises that introduced the concepts of *‘object orientation and aperture’* and *‘foreground and background awareness’*.

At the beginning of **day 2**, participants continued to explore meditative awareness of the environment with various exercises including a walking meditation in open awareness. Then the instructor asked participants to form small groups and share their personal experience of the weekend. After some time, the small groups gathered to share collectively the problems and difficulties which had emerged, so that the teacher could provide adequate feedback. Then the concepts of *‘empathy and compassion’* were introduced and the teacher asked participants to briefly cultivate feelings of self-compassion. After lunch, participants engaged in an exercise that involved switching between focused attention on, and open monitoring of, pain. The rest of the afternoon was spent further elucidating concepts of empathy and compassion, including an experiential exercise that presented images of people’s suffering to participants. Finally, during the closing meditation session, the importance of the intention to practice was discussed and emphasized. Participants were asked to fully engage in their own practice.

#### 2.2.2 Experiential exercises

Throughout the training weekend, subjects were prompted to familiarize themselves with the dimensions of subjective experience that were going to be of interest in the neuroscientific experiments. This familiarization was carried out by using experiential exercises. During each exercise, a dimension or process was introduced in a more or less explicit form. Some dimensions were experienced and described in the context of guided meditation sessions and teachings, while other exercises were implemented with the specific aim of familiarizing subjects with a phenomenological dimension. We refer the reader to the Supplementary Materials for a full description of these exercises.

At the end of the weekend, subjects received a document that briefly described each phenomenological dimension and reminded them how it was introduced by corresponding exercises during the weekend.

#### 2.2.3 Compliance and engagement with practice

The minimal goal of the training weekend was to give novice participants sufficient understanding and confidence to carry on practice autonomously, thus deepening their familiarity with the practices under study.

At the end of the weekend, participants received an explanation of what was expected of them in terms of homework practice. Participants were advised to practice for 20–30 min on a daily basis and to give equal importance to each of the three practices they had learned. In addition, they were asked to report the type and amount of practice in a practice logbook that was provided at the end of the meditation training weekend. In order to ensure truthful reports, participants were assured that non-compliance to these recommendations would not call into question their participation to the study.

Participants were provided with three 15 min-long audio recordings of guided meditations by their instructor to aid their practice (one recording for each of the three meditative practices). However, they were strongly encouraged to avoid relying exclusively on them and to get used to meditating unguided. Participants were also given excerpts from the book *Joy of Living*, summarizing most of the teachings received during the weekend: the physical posture, the mental attitude, as well as various meditative and experiential exercises examined during the weekend (Rinpoche & Swanson, 2007).

### 2.3 Practice metrics

**Fig. 1:**
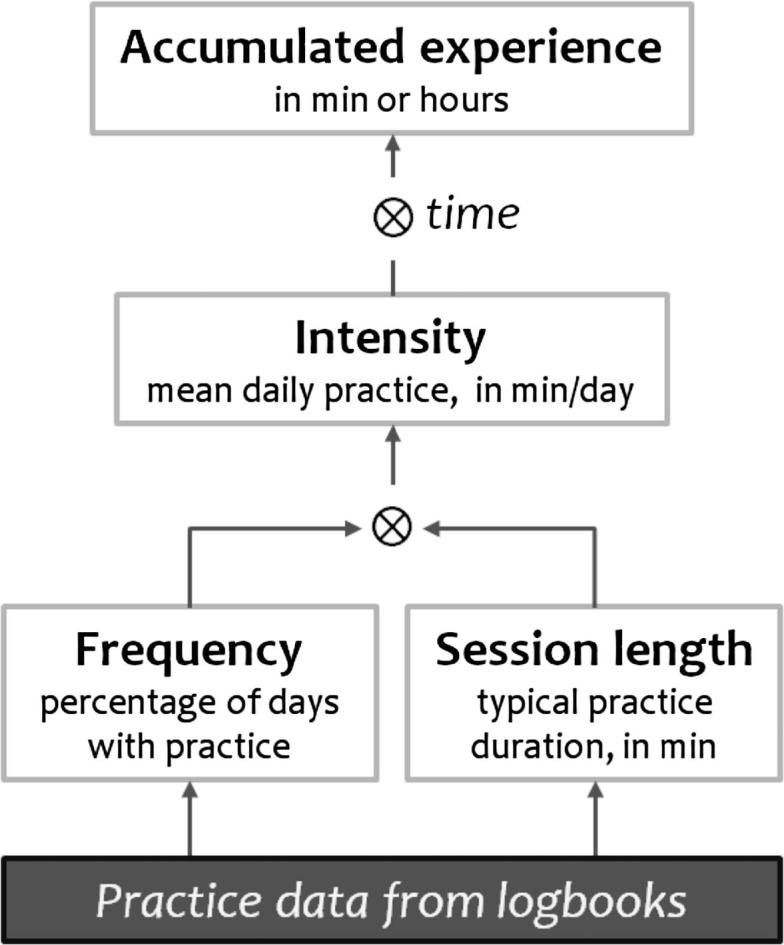
Four interrelated metrics were used to assess commitment to home practice.

Four metrics were used to assess participants’ home practice and degree of engagement: the proportion of days involving practice (hereafter referred to as *Frequency of practice* or simply *Frequency*); the average amount of practice during days with practice (hereafter, *Session length*); the daily average amount of practice, all days included (*Intensity of practice* or simply *Intensity*); and the total amount of practice (*Experience*).^3^ Fig. 1 describes how these four metrics relate to each other and how they were derived from the data contained in the practice logbooks. These metrics were also explored in relation to phenomenological ratings and behavioral measures from the experiments.

In addition, we calculated an index of balance between focus and open styles of practice, the Focus/Open Balance Index (hereafter referred to as FOB) for each participant. FOB is defined as the absolute difference between OM practice and FA practice, normalized by the sum of the two^4^:

$$1-\frac{\left|practice(FA)-practice(OM)\right|}{\mathrm{practice}(FA+OM)}$$

### 2.4 Protocol of the MEG experiment

**Fig. 2:**
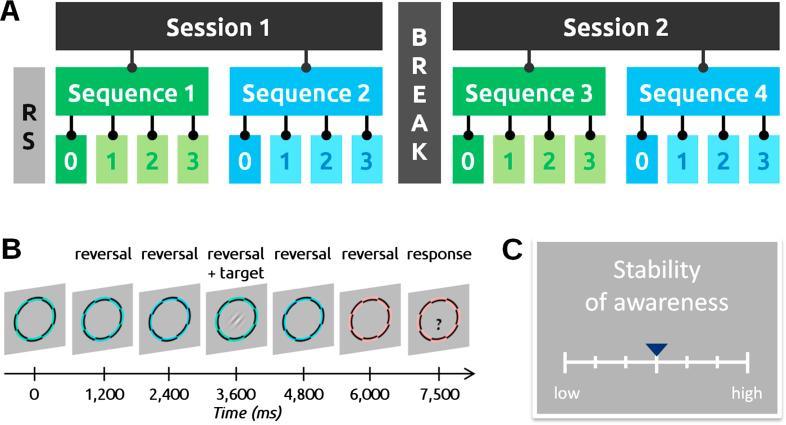
The neurophenomenological experiment MIMOSA, the self-report data of which is used in the current article. *A.* Hierarchical structure of the experiment. After an initial resting state period (RS), the experiment was divided into 2 sessions, with a 5–20 min break in between. Each session was divided into 2 sequences: one of FA (focused attention) and one of OM (open monitoring), presented in a randomized order. Therefore, there were 4 different combinations for the state order across the experiment: FA-OM-FA-OM (illustrated here), OM-FA-OM-FA, FA-OM-OM-FA, OM-FA-FA-OM; state order was counterbalanced across each group of participants. Each sequence consisted of 4 blocks: a first 7 min block of “meditation only” (block 0) followed by three ∼6 min long blocks of “meditation + task” with dynamic stimuli (blocks 1-2-3). During the “meditation only”, a white disk was displayed on a black background and participants were instructed to either use it as a support of their attention (in the case of FA) or to maintain their gaze on it (in OM blocks). During subsequent blocks, participants had to maintain the state induced in block 0, while going through 41 trials of a visual conscious report task. *B.* One trial of the task. During the task, a black-and-colored checkerboard was continuously displayed at the center of the screen. Each trial consisted of a series of checkerboard reversals, the last color of which was systematically deviant from the previous ones of the series (passive color oddball paradigm). A trial lasted 3–7 reversals. In 36 of the 41 trials, a Gabor patch set at threshold contrast was flashed for 50 ms, any time between the second and the last reversal. At the end of the trial, a question mark prompted the participant to report whether they had consciously seen it or not. *C.* After each of the 17 blocks of the experiment, participants rated 6 different dimensions of their experience using a Likert item.

One major purpose of the meditation course undertaken by novice participants was to train them in using phenomenological categories of interest in the study. In order to validate that they understood these categories as intended and used them appropriately, we analyzed the self-report data from a magnetoencephalographic (MEG) experiment with a hierarchical repeated measure design that included several periods of FA and OM meditations, along with a control (resting-state, RS) period (Fig. 2).

The experiment started with a staircase visual threshold calibration for a visual task whose detailed procedure and results are irrelevant for the present article but will be described in another publication. Following the calibration, we recorded a 7 min baseline period. Then, participants practiced FA and OM twice each, in sequences of approximately 24 min (Fig. 2a). Each sequence opened with a 7 min-long block of meditation only, during which participants were presented with a 1.5°-wide white dot in the middle of a black screen (Fig. 2b). The instruction was to keep the gaze steady on the white dot; in FA, participants were additionally instructed to use that disk as a support for the attention. This first block was followed by three blocks lasting approximately 5.5 min each, during which participants were instructed to maintain the meditative state while performing a simple visual conscious report task using a threshold stimulus embedded in a passive visual oddball paradigm (Fig. 2c). After each block, participants were invited to rate their experience over 6 different dimensions, using a 7-point Likert-type item (Fig. 2d): *Capacity* to apply the meditation instructions, *Stability* of the mind, *Clarity* of the mind, *Aperture* of the field of awareness (see table 1 for definitions), *Awareness of bodily sensations*, and *Wakefulness*. Here we will limit our analysis to the dimensions featured in the phenomenological matrix: Stability, Clarity and Aperture.^5^ Rating scales were thus introduced: *“Compared to your usual experience, how would you rate the last block in terms of Stability/Clarity/Aperture?”*

### 2.5 Statistical analyses

*Statistical modeling and inference.* ANOVAs were of type 2. Post hoc tests were performed using one- or two-sample *t*-tests, adjusted for family-wise multiple comparison using Tukey’s honestly significant difference (HSD) method. One-sample and paired two-sample tests were performed using the non-parametric Wilcoxon signed rank test. Linear mixed models were fitted using maximum likelihood and significance of fixed effects were evaluated using the likelihood ratio test. All linear regressions were ordinary least square (OLS) regressions.

*Correlation between scale ratings and variability of response times.* Outlier trials were defined as trials for which response times were not within 3 standard deviations from the mean value, for each participant, state and response type (*yes*/*no*), and excluded. An index of RT variability was defined for each block as the standard deviation of RTs. Finally, we computed for each participant the Pearson correlation coefficient between Stability ratings and RT variability. We did the same with Clarity. Correlation coefficients were z-transformed for the purpose of statistical modeling and are noted z hereafter. The data from one subject was removed because it had no variance in the Clarity scale (the subject responded 6 in all blocks).

*Model selection for multiple regression analyses.* For each of the effects related to phenomenological rating scores, we considered several potential predictors: metrics of home practice (*Intensity*, *Experience* and the *balance* between focus and open styles of practice), and an index of desirable responding (the score to the Balanced Inventory of Desirable Responding, *BIDR*). Practice data was missing for one participant, who was therefore excluded from subsequent analyses. We used an information-based model selection to determine the most important predictors for our data (Burnham & Anderson, 2002). Model selection is well suited to multiple regression analysis when the number of predictors is high compared to the number of data points; in addition, it virtually guarantees that no potential effect of interest is missed, as long as it is included in the variable set. We performed model selection in 2 steps, using *glmulti* for R (Calcagno & de Mazancourt, 2010). Firstly, we fitted all possible models that contained a subset of the predictors mentioned above and their two-way interactions, and that satisfied the marginality constraint (i.e. included all interaction terms as main effects). We used the corrected Akaike information criteria (AICc) as a measure of the quality of fit, because it is well adapted to small sample sizes (Hurvich & Tsai, 1989). Secondly, we selected models that were within 2 information criteria (IC) units of the best fitting model (Burnham & Anderson, 2002) for further consideration. Detailed results of the model selection output are presented in the Supplementary Materials. These include the *relative evidence weight*, a measure of relative importance of each term across the entire model space (Calcagno & de Mazancourt, 2010), comprised between 0 and 1.

## 3 Results

### 3.1 Structure of home practice

The total duration of participation to the entire study ranged from 41 to 163 days (99.4 ± 31.4 days) after the training weekend. Average daily practice (=Intensity) ranged from 1.3 to 30.5 min (15.9 ± 7.3 min; supplementary Fig. 1a, top left), suggesting that many participants fell short of the recommended amount of practice (20 to 30 min a day). However, when average daily practice was calculated over the number of days with at least *some* practice (rather than *all* days), the obtained Session length was found to range from 14.0 to 33.1 min (21.2 ± 5.5 min; supplementary Fig. 1a, bottom right). Participants dedicated 45.2 ± 16.8% of their practice time to OM (Open Monitoring), 33.4 ± 17.5% to FA (Focused Attention) and 21.4 ± 9.8% to CO (Compassion). A one-way repeated measure ANOVA revealed a significant difference between the proportion of time dedicated to the different practices (*F*(2,80) = 16.95, p < .0001, η^2^ = 0.30). Post hoc paired *t*-tests revealed that all comparisons of pairs of practices were significant (supplementary Fig. 1b).

Intensity of practice decreased linearly over weeks (supplementary Fig. 2a; R^2^_adj_ = 0.87, p < .001, β = -0.44, 95% CI [−0.53, −0.35]). A large portion of this drop (80.9%) is imputable to a sharp decline of Frequency of practice over weeks (supplementary Fig. 2b; R^2^_adj_ = 0.92, p < .001, β = −0.14, 95% CI [−0.16, −0.12]). The remaining 19.1% is due to a slight shortening of practice sessions (supplementary Fig. 2c; R^2^_adj_ = 0.22, β = −0.10, 95% CI [−0.18, −0.01]). Taken together, these results show that participants managed to follow the recommendation of 20-to-30 min-long practice sessions throughout the study, but failed to practice on a daily basis and were increasingly inclined to skip days.

### 3.2 Patterns in self-reports and relationships to practice

We tested three predictions that should be verified if the phenomenological self-reports are reliable. We examined whether the responses of the novices to the rating scales in the MEG experiment (i) were sensitive to the meditation state, in a way consistent with the known phenomenology of mindfulness practices (Lutz et al., 2015), (ii) exhibited classic temporal dynamics such as dose and fatigue effects, and (iii) were functionally informative, as would be suggested by correlations with behavioral measures.

In each instance, we tested whether desirable responding and/or features of participants’ home practice predicted the effects observed on self-reports. The results are summarized in Table 3.

**Table 3:** Summary of experimental results on self-reports of phenomenological dimensions during meditation states. All reported effects (discrimination of states, fatigue and discrimination of phenomenological dimensions) were associated to specific aspects of participants’ home practice. The importance of desirable responding score as a predictor was never higher than practice. DV: dependent variable; DC: demand characteristics; BIDR: Balanced Inventory of Desirable Responding; FA: focused attention; OM: open monitoring; FOB: focus/open practice balance index; Stb: Stability; Clr: Clarity; Apr: Aperture; RTV: response time variability.

DV	Interpretation	Level of DC	Predictors’ evidence across model space		
			BIDR	Practice	
${\mathrm{apr}}_{\mathrm{OM}}-{\mathrm{apr}}_{\mathrm{FA}}$	Discrimination of states	strong	0.31	0.55	FOB × Experience
$\frac{{\mathrm{stb}}_{3}-{\mathrm{stb}}_{1}}{2}+\frac{\mathrm{clr}-{\mathrm{clr}}_{1}}{2}$	Fatigue	moderate	0.40	0.74	Intensity_focus_
$z_{\mathrm{RTV},stb}-z_{\mathrm{RTV},clr}$	Discrimination of phenomenological dimensions	none	0.23	0.84	Experience

#### 3.2.1 Effect of states on self-reports

In the phenomenological model introduced in Lutz et al., 2015, Stability and Clarity are described as secondary qualities that are both increased when practicing either FA and OM (compared to mind-wandering), and even more so with expertise. In contrast, Aperture is hypothesized to increase specifically during the practice of OM.

**Fig. 3:**
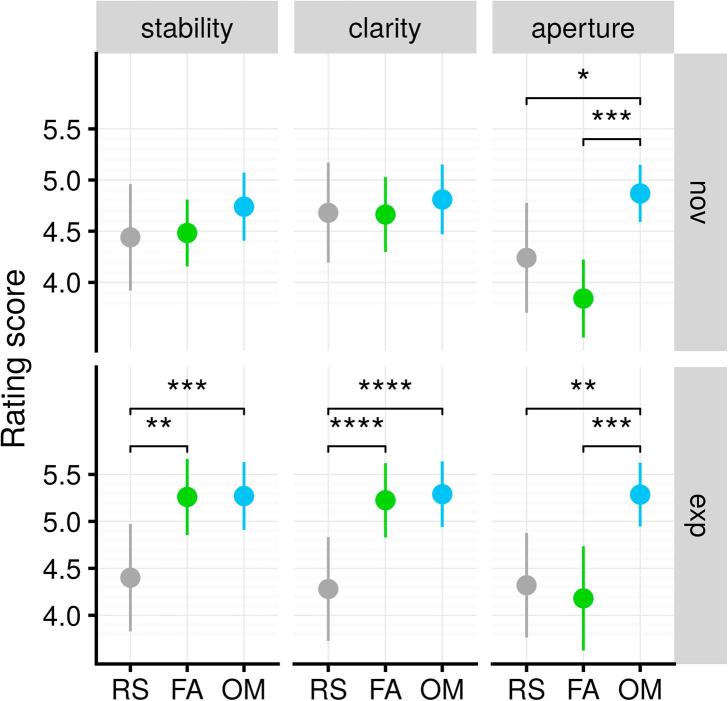
Effects of meditation states on three dimensions of experience reported by the participants. Both novice and expert groups reported greater Aperture of the attentional field during OM compared to RS and FA. Experts also reported greater Stability and Clarity during meditation compared to RS, but not novices. All ratings were given on a scale ranging from 1 to 7. RS: resting-state; FA: focused attention; OM: open monitoring. Errors bars are 95% confidence intervals. Significance levels: ^*^: p < .05; ^**^: p < .01; ^***^: p < .001; ^****^: p < .0001.

In order to test this prediction, we modeled the ratings per scale using a two-way ANOVA model with *state* (RS, FA, OM) as a within-subject factor and *group* (novices, experts) as a between-subjects factor (Fig. 3). For Aperture, we found a main effect of state (F(2,96) = 19.54, p < 0.001, $\eta_{G}^{2}$ = 0.15). Post-hoc *t*-tests showed that there was no significant difference between RS and FA (p > .32), while Aperture was reported significantly higher in OM than in FA and RS (p < .0001). For both Stability and Clarity, a small-to-medium state-by-group interaction was found (F(2,96) = 5.19, p = .007, $\eta_{G}^{2}$=0.027, and F(2,96) = 10.22, p < .001, $\eta_{G}^{2}$ = 0.045, respectively). Further post-hoc *t*-tests showed that experts’ ratings differed significantly in both dimensions between the control condition and each of the two meditation states (all p < .0001), but not between FA and OM (both p > .92). In contrast, there was no difference in neither Stability nor Clarity, across the 3 conditions, in the novice group (all p > .24). To summarize, participants’ ratings corroborated the hypothesized phenomenological pattern in the expert group for all three secondary dimensions tested, but only for Aperture in novices.

#### 3.2.2 Predictors of the state effect on Aperture

When participants are asked to rate the broadness of their attentional scope (i.e. Aperture) in two different meditation states referred to as “focus attention” and “open presence”, the fact that the expected response lies in the very names of the state conditions can hardly remain unnoticed. Thus, we cannot exclude the possibility that participants’ responses were influenced by their willingness to please the experimenter, and/or to show that they have correctly understood the meditation instructions.

Another, non-trivial hypothesis is that novice participants develop the ability to differentiate a state of focus attention and a state of broader awareness by getting equally familiar with both attentional styles. Said otherwise, we expect participants who have had more practice in OM than in FA to have more ease opening (or more difficulty narrowing) their attentional scope than participants who have developed an equal familiarity of the two styles of practice. As a result, we would expect the latter to better differentiate FA and OM on the Aperture scale than the former. The same reasoning can be straightforwardly applied, *mutatis mutandis*, to participants who favored FA over OM. We explored the plausibility of this hypothesis by testing whether our data were coherent with the ensuing predictions. We included an index of balance between FA and OM (FOB), along with other practice metrics (Intensity and Experience) and an index of desirable responding (BIDR) in the set of variables tested for model selection (see Section Methods, Statistical analyses).

Three models survived the model selection procedure: the best one included a significant FOB-by-Experience interaction (model A1), while the other two contained a significant FOB-by-Intensity interaction (models A2 and A3; see details in supplementary Table 1). BIDR was not present in any of these models, and its evidence across all models was found relatively low (0.31). The FOB-by-Experience interaction had a higher evidence across model space than the FOB-by-Intensity interaction (0.55 and 0.31, respectively). To illustrate how the balance between focus and open styles of practice interacts with the amount of practice, we performed a Johnson-Neyman post-hoc analysis of the interaction in model A1, using FOB as a predictor and Experience as a moderator. We found that for participants who had accumulated more than 23.9 h of practice, the FOB index positively predicts (p < .05) the self-reported difference in Aperture between FA and OM during the MEG experiment (see supplementary Fig. 3).

Considering that all our participants except one practiced OM more than FA at home, a higher FOB could have been entirely driven by FA practice in our dataset. Thus, the FOB-by-Experience interaction could actually hide an effect of Experience in FA only, which would lead to a very different interpretation of our results^6^. To test this alternative explanation, we fitted a model with Experience in FA as a single regressor, as well as a model that also included Intensity as a regressor. None of them was found significant (p > .98 and p > .16 respectively). We repeated this analysis with Experience in OM instead of FA – again, neither model was significant (p > .84 and p > .22, respectively).

To summarize, the results of our model selection analysis and post hoc tests are consistent with the hypothesis that equal familiarity with focus and open attentional states, rather than experience in any specific practice, drives the ability to differentiate between states.

#### 3.2.3 Temporal dynamics

The absence of effect on self-reported Stability and Clarity in novices over the experiment does not necessarily rule out the possibility that novice participants used these categories appropriately and informatively. For example, averaging ratings over the entire experiment could have occluded temporal effects. This is indeed what we have observed in our data (supplementary Fig. 4). We used linear mixed models to account for the nested nature of the MEG experiment structure (blocks within sequences within sessions). The models included all possible interactions in fixed effects, as well as random subjects’ intercepts and by-subject random slopes across blocks, sequences and sessions. We used two models: one for Stability ratings and one for Clarity ratings. In both models, we found an effect of block on ratings only for the first sequence of each session (i.e. sequences 1 and 3; cf. Fig. 2; Stability: χ^2^(3) = 8.58, p = .035; Clarity: χ^2^(3) = 7.85, p = .049), suggesting that the middle break had some sort of resetting effect. Post-hoc pairwise *t*-tests revealed a significant decrease of ratings from blocks 1 to 3 in sequences 3 and 4 (Stability: Δ = 0.62, 95% CI [0.19, 1.05], p < .002; Clarity: Δ = 0.43, 95% CI [0.09, 0.77], p < .007; all other p > .1), but no pairwise differences in other sequences (all p > .5).

#### 3.2.4 Predictors of the fatigue effect

The decrease of self-reported Stability and Clarity in novices after four blocks (∼24 min) of meditation may reflect fatigue. This is not surprising considering that most novices were not used to meditating for more than approximately 20 min during their daily home practice (see supplementary Fig. 1a). A corollary of this interpretation is that the longer and more frequently the participants were used to meditate, the less likely they should be to experience fatigue in the context of the experiment. We tested this prediction by modeling a fatigue index, defined for each participant as the difference between their ratings in block 3 and block 1 of sequence 1,^7^ averaged over the dimensions of Stability and Clarity. Five models were selected (supplementary Table 2). Intensity was present in 2 of them as a main effect, and in 2 others in interaction with BIDR. Surprisingly, FOB was present as a main effect in 4 out of the 5 selected models. Across all models, FOB and Intensity had the highest relative weighted evidence (0.74 and 0.66, respectively) closely followed by BIDR (0.60).

The fact that FOB was found as important as Intensity suggests that the mitigating effect of practice Intensity on Fatigue is driven by the level of engagement in a specific style of practice. To explore this idea, we performed a second model selection where we replaced Intensity and FOB by the two subcomponents of Intensity: Intensity_FA_ and Intensity_OM_, corresponding to the two styles of practice. For the sake of parsimony, we also removed Experience, which was already found to be of low importance. The only selected model from this new variable space had a single regressor, Intensity_FA_ (p = .034, β = 0.085, 95% CI [0.007, 0.162]; supplementary Table 3).

#### 3.2.5 Correlation with behavioral measures

Previous studies have reported intra-individual variability of performance (most notably response times) as a good predictor of whether the participant is on-task at a given moment or not (Bastian and Sackur, 2013, Seli et al., 2013). Based on this literature, we predicted that self-reported Stability, but not other dimensions, would be significantly correlated to variability of response times at the level of individual participants. We chose Clarity as a control dimension, for its similar pattern of sensitivity to state and group (see Fig. 3).

One sample Wilcoxon signed rank tests of z-transformed within-subject Pearson correlation coefficients against zero show that the RT variability correlated negatively with Stability (z = −0.26, 95% CI [−0.40, −0.12], p < .0003) as expected, but also with Clarity (z = −0.14, 95% CI [−0.28, −0.003, p = .046) (Fig. 5a, left). However, a two-sample paired test between z_stability_ and z_clarity_ was found significant (p = 0.041). Thus, even though self-reported ratings of Stability and Clarity were strongly correlated within subjects (Wilcoxon signed rank test on z-transformed correlation coefficients: z = 0.77, 95%CI [0.62, 0.94], p < .0001), the association with RT variability was significantly stronger for Stability than for Clarity. This suggests that although the phenomenological dimensions of Stability and Clarity tend to fluctuate naturally together, novices are able to differentiate them functionally in their reports, just like experts (Fig. 5a, right). In order to further assess the specificity of these findings, we repeated the same analysis using mean RT (instead of RT variability). We found that neither Stability nor Clarity correlated significantly with mean RT (both p > 0.1).

**Fig. 4:**
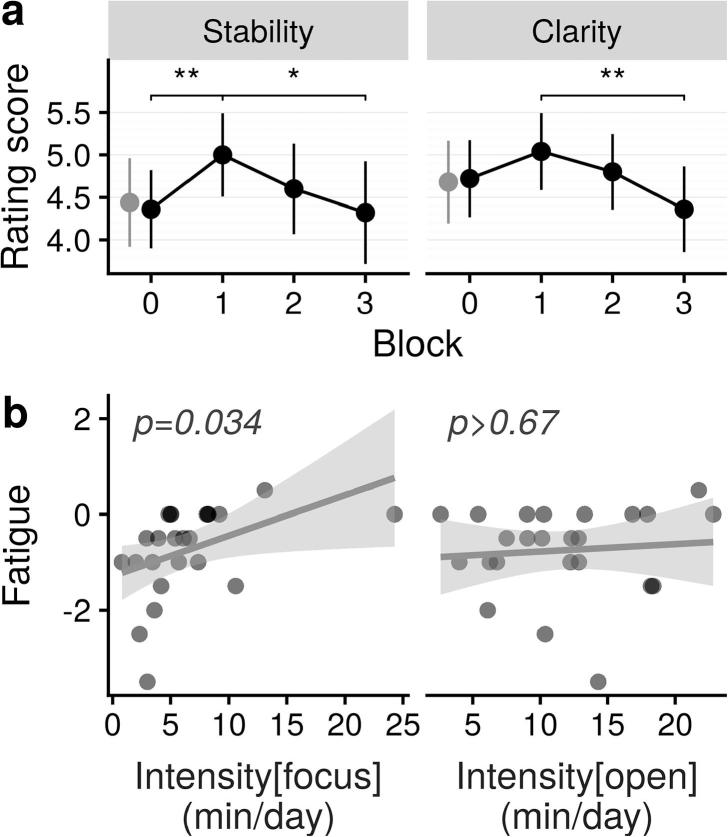
Evolution of self-reported Stability and Clarity in novices during the four blocks of the first meditation sequence. (a) There was a two-stage temporal pattern; namely, a boost of Stability from block 0 to block 1 (Δ = 0.64, 95% CI [0.03, 1.25], p = .036), followed by fatigue in subsequent blocks (Stability: Δ = −0.68, 95% CI [−1.29, −0.07], p = .023; Clarity: Δ = −0.68, 95% CI [−1.20, −0.16], p = .005). Ratings of the RS block are indicated in grey next to block 0. (b) This fatigue effect was reduced in those novices who engaged the most in focus (left) but not in open (right) styles of meditation. Errors bars are 95% confidence intervals. Significance levels: ^*^: p < .05; ^**^: p < .01.

**Fig. 5:**
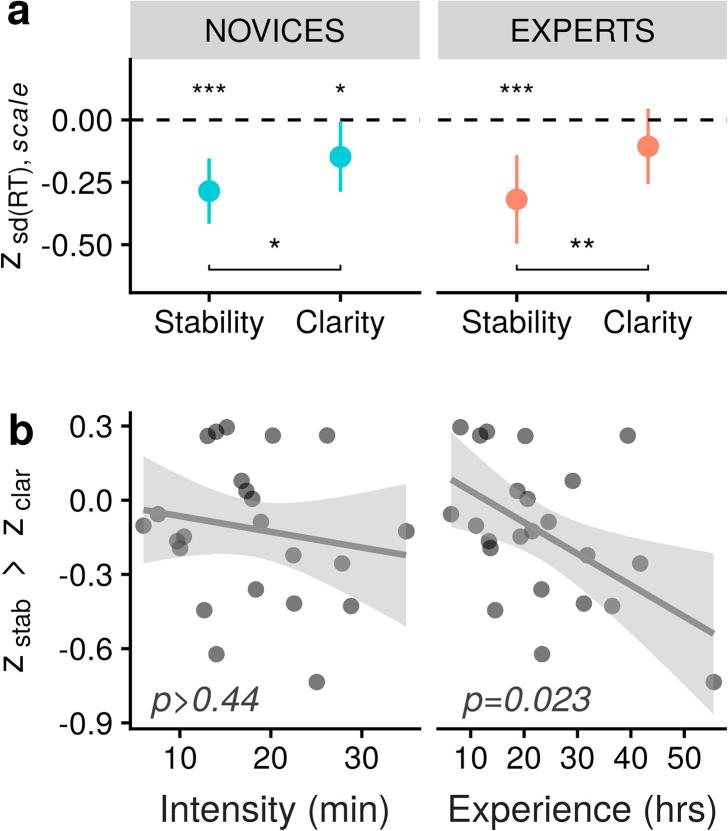
Self-reports from novices appear to be functionally relevant. (a) The variability of response time correlates negatively with self-reported stability. In novice participants, it also correlates negatively with clarity, but significantly less so, indicating that these two dimensions are properly differentiated by novices. (b) Functional differentiation of stability and clarity was higher in those novice participants with the highest amount of practice (right), while intensity of practice was not a reliable predictor (left). Errors bars are 95% confidence intervals. Significance levels: ^*^: p < .05; ^**^: p < .01; ^***^: p < .001.

#### 3.2.6 Predictors of the phenomenological specificity

Based on these results, we further hypothesized that this fine differentiation could have been implicitly trained in our novice group through the practice of meditation. Indeed, the reflexive quality cultivated in contemplative practices is expected to improve one’s familiarity with the specific phenomenal characteristics of different experiential dimensions. To test this prediction, we defined an index of phenomenological specificity as the difference between z_stab_, the z-transformed Pearson correlation coefficient between Stability ratings and RT variability, and z_clar_, the analogue measure for Clarity ratings. Only one model survived selection (supplementary Table 4), and it contained a single regressor, Experience (p = .023, β = −0.011, 95% CI [−0.021, −0.002]; Fig. 5b). Across all models, Experience had by far the highest evidence weight (0.84).

Following the suggestion of a reviewer, we further explored whether Experience in *particular practices* drove phenomenological specificity. We performed a second model selection using the amount of Experience in each practice (FA, OM and CO) and the 2-way interactions between these 3 variables (to model for potential synergies between practices). Three models were selected from this new variable space (supplementary Table 5). All of them included Experience_OM_ as a regressor but no interactions whatsoever. Experience_OM_ had by far the highest evidence weight (0.91), while Experience_FA_ and Experience_CO_ were on par (0.44 and 0.49 respectively).

## 4 Discussion

We described a meditation training protocol intended for naive candidates with no prior experience of meditation. We designed this protocol out of the need for a high-quality control group for a neurophenomenological study on the effects of meditation state and expertise in meditation on brain, behavior and physiology. The aim of the training was twofold: (1) to provide participants with sufficient background knowledge and direct experience with three types of meditations so that they could sustain a regular practice for an extended period of time, and be comfortable to practice in the laboratory context for the experimental tasks of the study; (2) to establish a common ground of relevant phenomenological categories with the participants, in order to allow them to report their experience during meditation states reliably.

Overall we found evidence that the phenomenological training was successful in the sense that participants’ self-reports: (i) were reliable and sensitive to the meditation state manipulation, (ii) exhibited expected temporal dynamics such as dose and fatigue effects, and (iii) were functionally informative; in addition each of these effects was more strongly predicted by the amount and structure of participants’ practice than by desirable responding as indexed by the BIDR questionnaire.

### 4.1 Motivation and compliance

In meditation research, motivation is often discussed for its potential confounding effect that limits the interpretability of longitudinal studies (Eberth & Sedlmeier, 2012). On the other hand, motivation of novice control participants can be considered a strength for the cross-sectional study of expertise as expert meditators, in so far as they dedicate a large of amount of time and resources to their training and practice, are expected to have strong motivation. Our study was highly demanding for the novices, as they had to engage in daily practice and participate in 6 to 8 experimental sessions over the course of several months. This, along with the multi-step recruitment procedure, acted as a filter for motivation.

The high level of motivation of the novice participants was reflected in the satisfactory level of commitment to the practice maintained throughout their participation to the study. Three months after their training, they were still accomplishing more than half of the prescribed amount of practice, in the absence of any reminders or booster sessions. This even though they were assured that dropping the practice would remain without consequences for their participation to the study and financial compensation. This laxity given to participants, while having the effect of revealing their intrinsic motivation, is not without shortcomings. Compassion meditation, for example, was largely neglected. This might be related to the dense set-up of our initiation program, which attempted to train the participants in three different forms of meditation in just two consecutive days. In contrast, the original *Joy of living* program on which the training was based requires that practitioners first engage in 6 months of regular practice before they can receive teachings on compassion. However, this shortcoming has limited consequences for our study as the goal was primarily to get participants accustomed to the concept and practice of compassion and sensitize them to its difference with empathic resonance.

A large majority (75%) of participants favored the practice of OM at home. This may come as a surprise considering that in many Buddhist contemplative traditions, OM practices are considered more advanced and are approached only after some training in FA (Lutz et al., 2008). However, this bias towards OM is consistent with the deliberate stance adopted by Mingyur Rinpoche, author of the *Joy of living* program, whereby one is invited to enter the field of open awareness from the outset.

### 4.2 Phenomenological proficiency

During their training, novice participants were introduced to phenomenological categories with the help of practical, experiential exercises. Using rating scales and behavioral data from one of the experiments to which they later participated, we have described three effects that can be interpreted as reflecting an effect of practice, phenomenal training, or both. Based on both *a priori* considerations and control for desirable responding, we have systematically assessed the potential confounding effect of demand characteristics and found limited support for it. We review the evidence (see also Table 3) and discuss other potential alternate interpretations below.

First, novice participants reported greater Aperture in OM compared to FA, just like experts, suggesting that they were able to distinguish the two practices. In addition, their responses on the Aperture scale was not driven by their diligence in any specific meditation style, but rather by the overall structure of their practice: the better they balanced focus and open styles of meditation, the larger the divergence in Aperture they reported (at least for participants who practiced the most: more than 24 h in total). This finding suggests that equal familiarity with different states is important for their optimal dissociation, at least at a beginner level. Future studies should test this hypothesis more rigorously using a longitudinal design with measures collected at baseline. Moreover, although we did not find any evidence for desirable responding in this data, we could not rule out the possibility that basic semantic priming inflated the reported dissociation in Aperture. Ideally, future training programs and experiments should try and avoid semantic overlap between meditation instructions and phenomenological dimensions altogether.

Second, self-reported Stability and Clarity had a two-stage dynamic during a series of 4 consecutive six-minute blocks of meditation, with a statistically significant decrease between the second and the last block. We interpret this phenomenon as a fatigue effect, rather than an effect of scale misuse. This is based on the observation that this decrease was negatively and specifically associated to participants’ Intensity of practicing focus attention. Interestingly, this specific association is consistent with the role of concentrative practices in Buddhist contemplative traditions. These practices are used as training to stabilize attention and other basic qualities such as clarity and effortlessness, before applying them to more advanced practices. However, this correlation is not necessarily indicative of an effect of training: it may be mediated by an individual trait (e.g. conscientiousness or stamina), present even before the meditation training, that could predict both sustained diligence in the practice of (the relatively effortful) focus attention, and endurance during meditation sessions in the MEG experiment. Regardless, the index of desirable responding was of lesser importance in comparison.

Third, we showed that participants’ ratings of the dimension Stability were functionally relevant, as they correlated with the variability of their response times. Using Clarity as a control dimension, we found that this functional relationship was specific. This finding suggests that participants were able to make fine distinctions between two close dimensions (this effect was true for both novice and expert practitioners). Regarding novices, we found that the more Experience they had at the day of the experiment, the sharper their phenomenological acuity. More specifically, we found that Experience in OM, compared to other practices, was the best predictor of this acuity. This is in line with the insight potential commonly attributed to OM-like practices in Buddhist contexts (Dahl, Lutz, & Davidson, 2015).

Here again, in the absence of longitudinal data, the correlation cannot be treated as direct evidence for a causal link involving learning. However, a noteworthy difference with the fatigue effect described above is that the practice metric that predicted phenomenological acuity (accumulated Experience) is not confounded with participants’ assiduity, because it depends as much on Intensity of practice as on the time elapsed between the training weekend and the experiment (which was variable and random across the group). Considering that Intensity of practice did *not* predict phenomenological acuity, it appears that a likely explanation of these results is that novice participants became more familiar with the phenomenal richness of their experience throughout the regular practice of meditation, and more proficient in reporting it with specificity and subtlety.

Taken together, our data support the claim that the novices in our study had some phenomenological literacy, and were able to report about qualities of their experience in an appropriate, meaningful and informative way, even though in some cases we could not conclusively rule out the possibility for an *additional* effect of demand characteristics.

### 4.3 Training and practice

We have introduced several metrics of practice beyond the oft-used *total amount.* Although these metrics are derived from each other, they are not entirely collinear. In particular, our study was able to dissociate Experience (=total amount of practice) from Intensity (=daily average of practice) by having a large variability in the time elapsed between the training and the MEG experiment, across the novice group (from 27 to 133 days; *M* = 79, *SD* = 29). Moreover, we showed that these two metrics could be functionally dissociated when correlated with subjective ratings or behavioral measures. Such dissociations could point to potentially different mechanisms of trait changes brought about by the practice of meditation. This observation is consistent with the finding that intensive retreat practice, but not routine daily practice is associated with reliable differences in resting respiration rate in experienced meditators (Wielgosz, Schuyler, Lutz, & Davidson, 2016). It is also reminiscent of the work of Antonova, Chadwick, and Kumari (2015) who found that intensity of practice was a better predictor of decreased habituation to the acoustic startle reflex than total hours of practice. Future investigation of the mechanisms of meditation would benefit from a systematic exploration of various practice metrics and their relation to experimental outcomes, for both novice and expert practitioners.

What is the minimum amount of practice that should be required from novice participants for the quality of their phenomenological self-reports to match those of experts’, on the dimensions explored here? Based on our experimental results, we can provide tentative, rough estimates. In our samples of participants, 20–40 h were necessary for novices to reach a level of phenomenal specificity comparable to the one of experts; a minimum of 20 min of practice per day on average and a high balance between practices (no more than 40% bias) enabled novice participants to differentiate different styles of meditation as well as expert practitioners. All these criteria are much higher than what most past cross-sectional studies of meditation expertise have required from their control participants (Fox et al., 2016), but are sufficiently low to be practically accessible and implemented in future studies.

### 4.4 Self-rating scales

We used self-rating scales as tools to help participants translate qualities of their lived experience into quantities that can be manipulated, transformed and statistically analyzed just like any other numerical measure. Such tools raise vexed issues: for example, how can we know that participants use the scales as intended? How can we know that participants, or groups of participants, are not construing a given scale in widely different ways? How can we even be sure that a given participant is consistent in the way he/she uses a scale over time or across experimental conditions, for that matter? To take the example of stability of meditation states, we may argue that stability refers to qualitatively different experiences in FA and OM. In FA, stability reflects the sustained focus on a given object and therefore the stability of mental content. In contrast, OM stability reflects the absence of grasping and as such, should not be affected by variations in content.

Our approach of phenomenological training pragmatically addresses the issue of interpretation by mapping linguistic definitions of categories onto features of lived experience induced and revealed by simple experiential exercises. Performing these exercises in the context of a group, under the guidance of an instructor and with the possibility to share their understanding and reflect collectively, has the potential to attenuate idiosyncratic apprehensions of the phenomenological categories. In addition, concerns related to the subjectiveness and incommensurability of self-reported ratings were pragmatically addressed using within-subject designs and analyses.

Even if our results suggest that our methodological approach was effective in detecting phenomenological fluctuations, it is worth mentioning the low variance of our self-rating data. As an example, 41 out of 50 of our participants used only three values out of the seven available in the Stability and Clarity scales; for Clarity, 18 out of 50 participants used only two values. This suggests a limitation of our experimental design and/or our training program. For instance the relatively short duration of laboratory experiments may not be sufficient to experience large fluctuations in these dimensions. Finer rating scales could be used as a compensation to increase data variance. Another possibility is that our training program was insufficient in developing participants’ fine-grained sensitivity to these scales. Further methodological work is needed to address these limitations.

### 4.5 Future directions

The role of meditation practice for cognitive science was extensively discussed by Varela et al. (1991), becoming a part of their ‘enactive’ approach and then of Varela’s neurophenomenological program (Varela, 1996). We have provided preliminary evidence that meditation experience improves the reliability of self-report data by improving the functional specificity of self-reports, and by shielding them from the effect of demand characteristics. However, several questions remain open and should be addressed by future research.

First, the impact of training on the quality of first-person data should be more rigorously assessed using high-quality, longitudinal, randomized controlled trials. In particular, future work should tackle the open question of whether specific phenomenological training such as the one we implemented through experiential exercises is required to improve the quality of first-person data, or whether meditation practice is sufficient in itself.

In order to evaluate the confounding effect of demand characteristics on our first person-data, we have used an index of desirable responding. Unfortunately, the validity of the questionnaires designed for this purpose, including the one used in this study, has been frequently questioned as they are unable to distinguish between genuine personality traits and self-enhancement (Paulhus, 2002). Special attention should be given to more recent efforts to overcome these limitations using alternative and potentially complementary approaches (Kwan et al., 2004, Paulhus et al., 2003).

We have provided evidence for reliable first person reports of the phenomenology of meditation experience. However, the generalizability of the phenomenal insight provided by meditation practice to other, non-meditation-related applications remains disputed (see Khalsa et al., 2008 for an example of negative result) and warrant more research.

Rather than provide a standardized, validated, ready for use protocol, our intention was to raise methodological concerns pertaining to the quality of control groups used in cross-sectional studies of meditation, and to argue for the possibility of obtaining informative experiential self-reports from adequately trained participants. Although we described in detail the protocol that we designed to address these issues, including the experiential exercises used for the phenomenological training of the participants, our approach is tailored to the specific context of our study, and to a particular phenomenological model of meditation among others (Bodhi, 2011, Lindahl et al., 2017, Van Dam et al., 2018). Still, we hope that the process, rather than the content, will inspire researchers in the field to further explore these critical issues.

## Ethics

The entirety of the Brain & Mindfulness project, including the meditation training weekend and the MEG experiment, was approved by the local ethics committee (CPP Sud-Est III, authorization number 2015-A01472-47). All participants signed an informed consent prior to their participation to the meditation training.

## Footnotes

1 Such techniques were sometimes dubbed ‘second person’ methods, because they rely on the critical interventions of an interviewer.

2 One study by Lutz et al. (2009) included samples from two other studies in the *meta*-analysis and was not taken into account. We excluded studies on yoga and chanting.

3 Capitals are used to distinguish these terms from their common definitions.

4 Note that for the calculation of FOB, using Intensity or Experience as measures of practice produce exactly the same result because, for each participant, Intensity is equal to Experience divided by a time factor (the number of days between the training week-end and the experiment). This factor would be cancelled out in the ratio present in the formula of FOB.

5 Just like for practice metrics, capitals are used for phenomenological dimensions to distinguish the terms from their common definitions.

6 We thank an anonymous reviewer for suggesting this alternative interpretation.

7 The same analysis based on combined data from sequences 1 and 3 leads to the same conclusions.
