# Supplementary material for: Training novice practitioners to reliably report their meditation experience using shared phenomenological dimensions
Source: Conscious Cogn. 2019 Feb;68:57–72. doi: 10.1016/j.concog.2019.01.004 (PMC6374282; doi:10.1016/j.concog.2019.01.004)
Supplement: Supplementary data 1 [file mmc1.docx]

#

## Training novice practitioners to reliably report their meditation experience using shared phenomenological dimensions

*Consciousness and Cognition*

Oussama Abdoun

Jelle Zorn

Stefano Poletti

Enrico Fucci

Antoine Lutz*

Lyon Neuroscience Research Centre, INSERM U1028, CNRS UMR5292, Lyon 1 University, Lyon, France

*Correspondence concerning this article should be addressed to Antoine Lutz:
antoine.lutz@inserm.fr

# Supplementary material

[Experiential exercises implemented in the phenomenological training protocol 2](#_Toc534898194)

[Characteristics of novices’ practice 4](#_Toc534898195)

[Model selection for self-reported phenomenological effects 5](#_Toc534898196)

[Temporal dynamics of Stability and Clarity 6](#_Toc534898197)

[Other data 7](#_Toc534898198)

[References 7](#_Toc534898199)

## Experiential exercises implemented in the phenomenological training protocol

**Effort**

The dimension of effort was introduced in the context of practicing Focused Attention on the sound of a bell. The teacher asked the participants to experience the difference between concentrating on a specific sound while preventing the mind from wandering and simply recognizing the same sound in the field of awareness (“just listen to the sound”).

**Absorption and Meditative Awareness**

The difference between being absorbed in a mental phenomenon and being aware of it and its nature has been described during teachings throughout all the course of the training weekend. In particular, participants have been invited to recognize the switch between absorption and awareness that occurs at the very moment they realize their mind has wandered away during meditation.

**Object Orientation and Aperture**

In the context of a guided practice, subjects were asked to focus their attention over a small visual object (a flower, a piece of tissue, etc.). Subjects were first asked to concentrate on a specific detail of the object, then on the entire object and finally on the entire visual field. This exercise was implemented to introduce the concepts of object orientation and aperture with a direct experience of the different degree of attentional focus in a given sensory modality, and the aperture of the field of awareness.

**Foreground and Background Awareness**

During the weekend, participants were guided through a meditation practice using physical sensations as support and then shifting the attention over the visual field. Participants could experience that, although their attention shifted and changed the main object of focus, physical sensations that were previously in the “foreground” could still be experienced in the “background”.

**Empathy and Compassion**

During the second day of the training weekend, subjects were introduced to the processes of empathy and compassion with the help of an experiential exercise. The aim of this exercise was to help the subject experiencing and understanding the practical meaning of compassion, as intended in the Buddhist framework. The access to this understanding and experiential testing was fostered by the contrasting experience of empathy, based on the process of eidetic reduction and imaginative variation (Husserl 1991).

In practice, two highly emotional pictures, depicting people suffering, were shown to the subjects. The pictures were taken from the Internal Affective Picture System database (IAPS, Lang et al. 1997) and selected based on negative valence and high arousal. Subjects were warned that the pictures were characterised by a strong emotional content. They were first asked to look at the picture without any instruction (for about 3 minutes). Subsequently, they rested their mind for some minutes and shared their experience of looking at the pictures. The teacher acknowledged the subjects’ experiences and introduced the concept of empathy and the practice of compassion.

Subjects were asked to look at the pictures again and were guided by the teacher to empathize with the sufferer with sentences such as: “the person suffers and you suffer with her”. They were also prompted to silently repeat sentences such as: “I feel your sufferance” or “Your sufferance is my sufferance”. Moments of “empathy training” were intermixed with the practice of open awareness. Subjects were then guided through a practice of compassion, using the same pictures as support. This practice followed the form of compassion meditation as implemented in the secular program of the Buddhist tradition on which the training was based. In this tradition, feelings of compassion are aroused through the mental visualization of close and distant suffering beings. Once a felt experience of the desire to help is established, the visualization support may be dropped and the practitioner simply rests his/her awareness into the compassionate feelings.

Finally, participants were encouraged to share their thoughts and comment on the difference between the two experiential exercises and the processes of compassion and empathy, as well as their understanding of these processes before and after the exercise.

**Clarity**

The dimension of clarity was introduced and described to the participants at the end of the weekend. They were asked to recall how clear their mind was when undergoing the thermal pain threshold calibration, compared to trying to use drowsiness as a support for meditation. The practice on drowsiness was proposed after lunch on the weekend days. This exercise aimed to help novices familiarizing with different degrees of clarity of their mental experience, which can partly depend, at least at an early stage of the meditation practice, on physiological states.

##

## Characteristics of novices’ practice


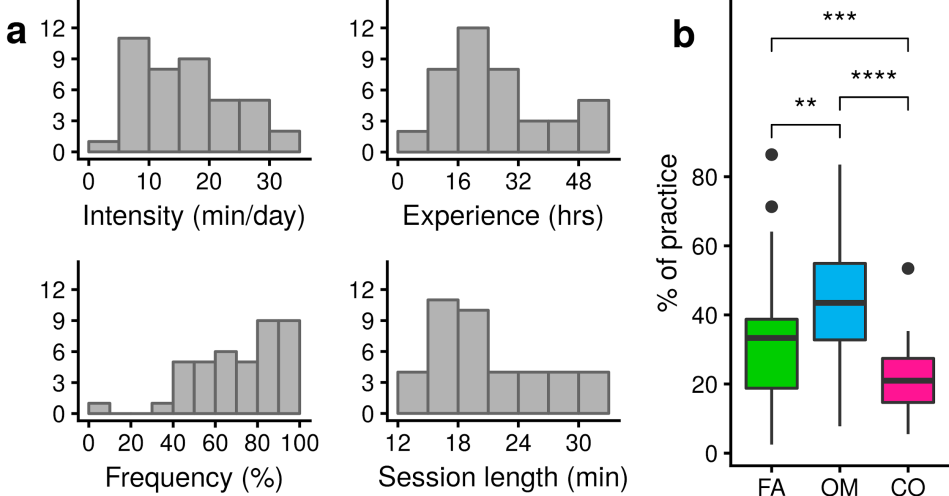


**Supplementary Fig. 1** Distributions of the four metrics over the group of novice participants. Participants were asked, but not required, to meditate daily for 20 to 30 minutes for the whole duration of the study, which ranged from 6 to 23 weeks. (**b**) Participants favored the practice of Open Monitoring (OM) over Focused Attention (FA), and tended to neglect the practice of Compassion (CO). We did not find any effect of type of practice (FA, OM, CO) on Session length (one-way repeated measure ANOVA: *F*(2,80)=1.47, p=.23). In order to determine whether individual preferences towards a given practice were present at the outset or emerged over time, we used the percentage of weekly practice time dedicated to the preferred practice as a measure of practice bias. Thus defined, practice bias ranges from 33.3% (no bias: the participant dedicates the same amount of time to each of the three practices) to 100% (full bias: the participant engages in only one practice). Practice bias was found high already in the first week after the training weekend (M=59%, SD=16%), and increased linearly over weeks (R²_adj_=.27, p<.016, β=.46, 95% CI [.10, .81]; data not shown). Significance levels: *: p<.05; **: p<.01; ***: p<.001


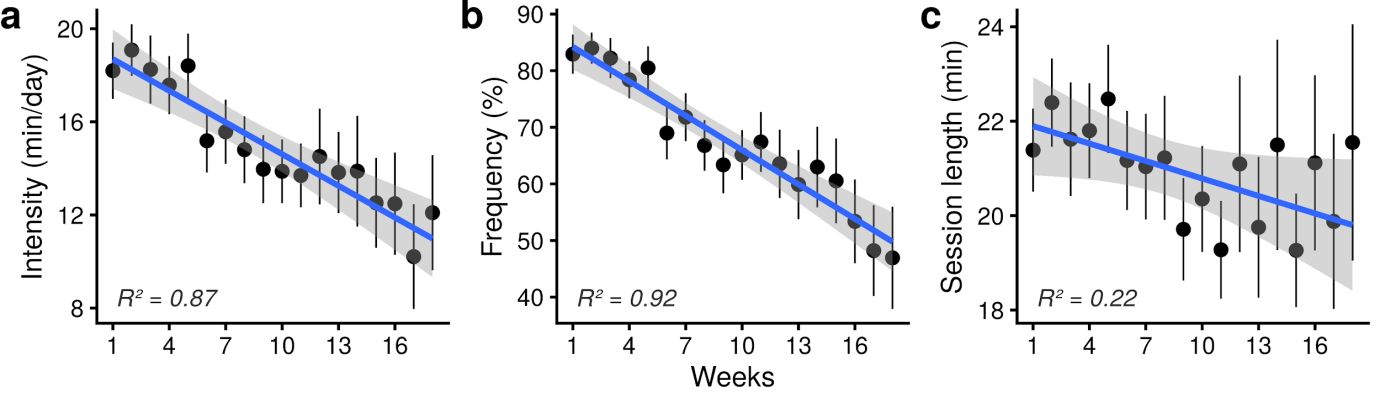


**Supplementary Fig. 2** The amount of daily practice decreased steadily over time (**a**). A closer look reveals that this effect is mostly due to a decline of the Frequency of practice (**b**) rather than a shortening of Session lengths (**c**). Errors bars are 95% confidence intervals.

## Model selection for self-reported phenomenological effects

Presented below are the detailed results of each of the model selections performed. The following information is provided for each model: the variables that compose it, the difference between the AICc of the model and the AICc of the best model (ΔIC), the variance explained by the model (R²_adj_), the global level of significance of the model (p), and the p-value of each of the model’s terms (main effects and two-way interactions, if any; bold: <.05; italics: <.1; n.s.: >.1). In addition, we provide the *relative evidence weight*, a measure of relative importance of each term across the entire model space (Calcagno and de Mazancourt 2010), comprised between 0 and 1.

| **model #** | **ΔIC** | **R²_adj_** | **p** | **BIDR** | **INT** | **EXP** | **FOB** | **FOB:INT** | **FOB:EXP** |
| --- | --- | --- | --- | --- | --- | --- | --- | --- | --- |
| A1 | 0 | .38 | **.010** |  | **.007** | n.s. | *.09* |  | **.007** |
| A2 | 1.13 | .25 | **.048** |  | **.04** | n.s. | n.s. | *.053* |  |
| A3 | 1.44 | .27 | **.025** |  | *.07* |  | n.s. | **.033** |  |
| **Relative evidence weight** | | | | .31 | **.85** | .66 | **.86** | .35 | .55 |

**Supplementary Table 1** Models selected for the state effect on Aperture

| **model #** | **ΔIC** | **R²_adj_** | **p** | **BIDR** | **INT** | **EXP** | **FOB** | **BIDR:INT** | **BIDR:EXP** |
| --- | --- | --- | --- | --- | --- | --- | --- | --- | --- |
| B1 | 0 | .34 | **.016** | n.s. | n.s. |  | **.007** | **.017** |  |
| B2 | 0.90 | .17 | **.050** |  | *.066* |  | *.071* |  |  |
| B3 | 1.69 | .45 | **.010** | n.s. | n.s. | n.s. | **.004** | **.003** | *.056* |
| B4 | 1.81 | .08 | *.098* |  | *.098* |  |  |  |  |
| B5 | 1.97 | .07 | n.s |  |  |  | n.s. |  |  |
| **Relative evidence weight** | | | | .60 | .66 | .34 | .74 | .36 | .10 |

**Supplementary Table 2** Models selected for the fatigue effect

| **model #** | **ΔIC** | **R²_adj_** | **p** | **BIDR** | **INT_FA_** | **INT_OM_** |
| --- | --- | --- | --- | --- | --- | --- |
| C1 | 0 | .15 | **.034** |  | **.034** |  |
| **Relative evidence weight** | | | | .40 | .74 | .34 |

**Supplementary Table 3** Models selected for the fatigue effect, after differentiating Intensity_focus_ and Intensity_open_.

| **model #** | **ΔIC** | **R²_adj_** | **p** | **BIDR** | **INT** | **EXP** | **FOB** |
| --- | --- | --- | --- | --- | --- | --- | --- |
| D1 | 0 | **.19** | **.023** |  |  | **.023** |  |
| **Relative evidence weight** | | | | .23 | .30 | **.84** | .21 |

**Supplementary Table 4** Model selected for phenomenological specificity, defined as the difference between correlations between variability of reaction time on one hand, and Stability and Clarity on the other hand.

| **model #** | **ΔIC** | **R²_adj_** | **p** | **EXP_FA_** | **EXP_OM_** | **EXP_CO_** |
| --- | --- | --- | --- | --- | --- | --- |
| E1 | 0 | **.28** | **.006** |  | **.006** |  |
| E2 | 1.22 | **.29** | **.012** |  | **.005** | n.s. |
| E3 | 1.48 | **.35** | **.011** | n.s. | **.011** | **.***063* |
| **Relative evidence weight** | | | | .44 | **.91** | .49 |

**Supplementary Table 5** Results of post-hoc model selection for phenomenological specificity.

## Temporal dynamics of Stability and Clarity


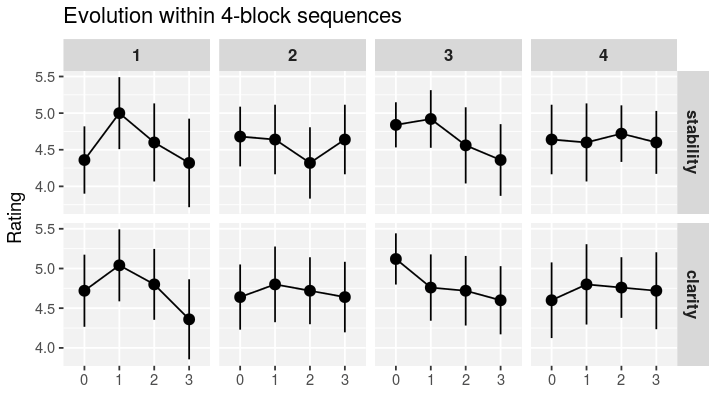


**Supplementary Fig. 4** The temporal dynamic of Stability and Clarity ratings in novices across 16 blocks divided into 4 sequences (see fig. 2). All ratings were given on a scale ranging from 1 to 7. Error bars = 95% CI.

## Other data

|  | **BIDR** | **Intensity** | **Experience** |
| --- | --- | --- | --- |
| **Intensity** | **0.431** * [0.034, 0.711] |  |  |
| **Experience** | 0.131 [-0.287, 0.508] | **0.617** ** [0.284, 0.817] |  |
| **FOB** | 0.197 [-0.224, 0.556] | -0.061 [-0.454, 0.351] | -0.058 [-0.451, 0.354] |

**Supplementary Table 6** Pearson cross-correlations between variables of interest. Values between square brackets are 95% confidence intervals. Values in bold are significant. Significance levels: *: p<.05; **: p<.01

**
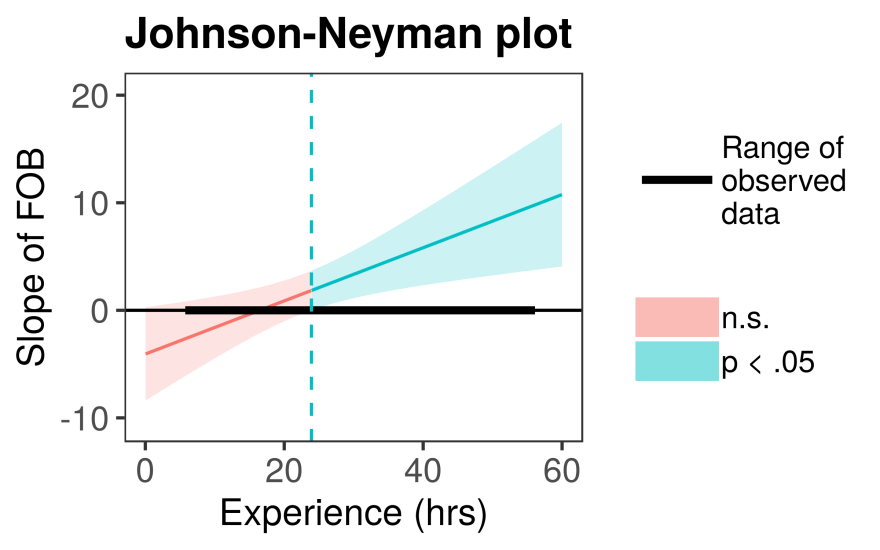
**

**Supplementary Fig. 3** Johnson-Newman plot of the FOB-by-Experience interaction in model A1 of the state effect on Aperture.

## References

Calcagno V. and de Mazancourt C., glmulti: An R package for easy automated model selection with (generalized) linear models, *Journal of Statistical Software* 34 (12), 2010, <https://doi.org/10.18637/jss.v034.i12>.

Husserl, E. (n.d.). On the phenomenology of the consciousness of internal time (1893–1917), 1991st ed. Kluwer Academic Publishers.

Lang P.J., Bradley M.M. and Cuthbert B.N., International affective picture system (IAPS): Technical manual and affective ratings, *NIMH Center for the Study of Emotion and Attention* 1997, 39–58.
